# Impact of a Home-Based Remote Patient Monitoring System on Hospitalizations and Emergency Department Visits of Older Adults With Polypathology: Multicenter Retrospective Observational Study

**DOI:** 10.2196/64989

**Published:** 2025-09-10

**Authors:** Damien Testa, Vincent Iborra, Mireille Dutech, Manuel Sanchez, Agathe Raynaud-Simon, Elise Cabanes, Christine Chansiaux-Bucalo

**Affiliations:** 1 EPOCA U&I Nanterre France; 2 Department of Geriatrics AP-HP North, Bichat, Beaujon and Bretonneau Hospitals Paris France; 3 Centre Hospitalier Rives de Seine Courbevoie France

**Keywords:** older adult, telemedicine, telemonitoring, tele-expertise, home-based medical care, hospital readmission, patient care coordination, hospitalization reduction, polypathology, geriatric

## Abstract

**Background:**

Every year in France, 40% of people aged ≥80 years are hospitalized, with an average length of hospital stay of 25 days and a readmission rate of 14% to 30% within the month following discharge. This situation is putting pressure on the health care system, encouraging the reinforcement of home care to reduce avoidable hospitalization. The EPOCA remote patient monitoring (RPM) system is a medical and social telehealth solution specialized in RPM, teleconsultation, tele-expertise, and care coordination in emergency medicine and geriatrics. The platform provides long-term medical support at home (MSAH) with 24-7 telemonitoring of older adults with polypathology. We hypothesized that receiving long-term MSAH via the EPOCA RPM system would be associated with a reduction in the rates and durations of hospitalizations or emergency department (ED) visits in older adults with polypathology.

**Objective:**

We aimed to compare the hospitalization and ED visit rates, as well as the cumulative hospital stay duration, before and after enrollment in the EPOCA RPM system for older adults with polypathology.

**Methods:**

This retrospective observational study included older adults (aged ≥70 years) with polypathology (>2 affected systems) followed throughout 2 different types of long-term MSAH between February 2022 and October 2023. We compared the number of hospital admissions, including ED visits; the cumulative duration of hospital stays during the follow-up; and the average length of hospital stays during the period corresponding to the MSAH program (Y) compared to the year before entering the program (Y – 1). Subgroup analyses were conducted according to the severity of the participants’ disability.

**Results:**

A total of 120 participants were included in the MSAH program, with a mean age of 86.8 (SD 7.9) years. Hospitalization and ED visit rates decreased (−48%) between the Y – 1 and Y periods, as did the total duration of hospital stays (−63%). A significant reduction in number of hospitalizations (median decreased from 1.0 to 0.0 per patient per year; *P*<.001) and ED visits (median decreased from 1.0 to 0.0 per patient per year; *P*<.001) was observed between the Y – 1 and Y periods. This corresponded to a significant median reduction of 14 days spent at the hospital per patient per year (*P*<.001). The decrease in hospitalization and ED visit rates and numbers was greater in participants with severe disabilities than in those with no or moderate disabilities.

**Conclusions:**

Among older adults with polypathology, the EPOCA RPM system is associated with reduction in number of hospitalizations, ED visits, and duration of hospital stays. Facing the challenge of population aging, home telemonitoring embedded in the health care system offers potential benefits.

## Introduction

### The Challenge of a Health Care Pathway for Older Adults

The aging of the population is a global phenomenon challenging the health care system [1,2]. Life expectancy at birth continues to rise worldwide, and in Europe, it stands at approximately 85 years for women and 80 years for men [3]. For instance, in France, on January 1, 2024, a total of 28% of the French population (18.5 million people) was aged ≥60 years, and 6.1% (4 million people) were aged ≥80 years [4]. Concomitantly, the proportion of older adults with disabilities is growing [5].

In this context, the health care system faces multiple challenges as the supply of medical personnel does not increase proportionally to the demographic growth in the older population. Demand for medical services and specialized home care for older adults is particularly growing [6]. At the same time, the overall number of physicians is lower in France than the European Union’s average (3.2 vs 4.1 physicians per 1000 population in 2021, respectively). In particular, the number of general practitioners (GPs) decreased over time by 8% between 2012 and 2022 [7]. Due to work overload, more and more GPs are adapting their practice toward refusing new and occasional patients or appointments at home [8,9]. Thus, in 2021, approximately 6% of people aged ≥80 years did not have an assigned GP, and 10.6% of them were people with long-term illnesses [10].

In addition, hospitalization is often the quickest and commonest way to respond to the complexity of managing older adults with multiple pathologies [11,12], causing organizational challenges, especially for emergency departments (EDs). While they represent 6.1% of the French population, people aged ≥80 years accounted for 12.5% of persons hospitalized in 2018, and 40% of people aged ≥80 years experienced at least one hospital stay during the year, with an average length of hospital stay per patient of approximately 25 days [13,14]. Furthermore, 13% to 40% of admissions to EDs and >30% of hospitalizations are believed to be avoidable [15].

Early readmission after a hospital stay is a frequent occurrence involving between 14% and 30% of patients within the month following discharge [16-18]. Telemonitoring through the use of telemedicine is a way to optimize care for older adults with multiple chronic diseases [19-21] and could lead to a significant reduction in hospital readmissions and lower health costs [22-24]. Remote patient monitoring (RPM) is a telehealth activity to collect and analyze patient health indicators or medical data with the objective of ensuring that patients are safely monitored, improving patient outcomes and reducing avoidable hospital admissions (see the reviews by Farias et al [25], Le Bras et al [26], and Taylor et al [27]).

A meta-analysis of integrated care programs using telemedicine in patients with chronic illnesses (chronic heart failure, diabetes mellitus, chronic obstructive pulmonary disease, and asthma) reported beneficial effects, including reduced use of health care resources (such as hospital admissions and readmissions) [21]. Most mentioned studies focused on 1 chronic condition, whereas older adults usually experience polypathology. More recently, another meta-analysis concluded that transitional care interventions with telemedicine were associated with a 40% reduction in hospital readmissions in older patients (aged ≥65 years) who were chronically ill and had a high risk of readmission (including polypharmacy or polypathology) [13]. The studies included in this meta-analysis reported telehealth assistance mostly performed via phone calls or videoconferencing. Moreover, the ED visit rate did not decrease, suggesting a lack of coordination of community care services when health problems were detected. Results from a French multicenter observational study also evidenced the potential effectiveness of geriatric expertise phone hotlines in preventing unnecessary hospital admissions (36.3% decrease) to the ED or a geriatric acute care unit [28]. The eCOBAHLT (Elderly Chronic diseases Online Biometric Analysis Home Living Technology) randomized controlled trial assessed the impact of a home-based telesurveillance program on hospital readmissions in older patients (aged ≥65 years) with at least 2 comorbidities and discharged home after hospital care. The study found a significant reduction in readmissions for the intervention group (40.4%) compared to those receiving standard care (48.7%) during a 12-month follow-up [29]. However, as in previous studies, RPM generated more ED visits without hospitalizations. The impact of RPM on increasing ED visits may be an important issue. Integrating RPM into a program involving coordination of care between hospital and community services may help reduce unnecessary ED visits and hospitalizations.

### The EPOCA RPM System Included in the Vigie-Age Project

**Figure 1:**
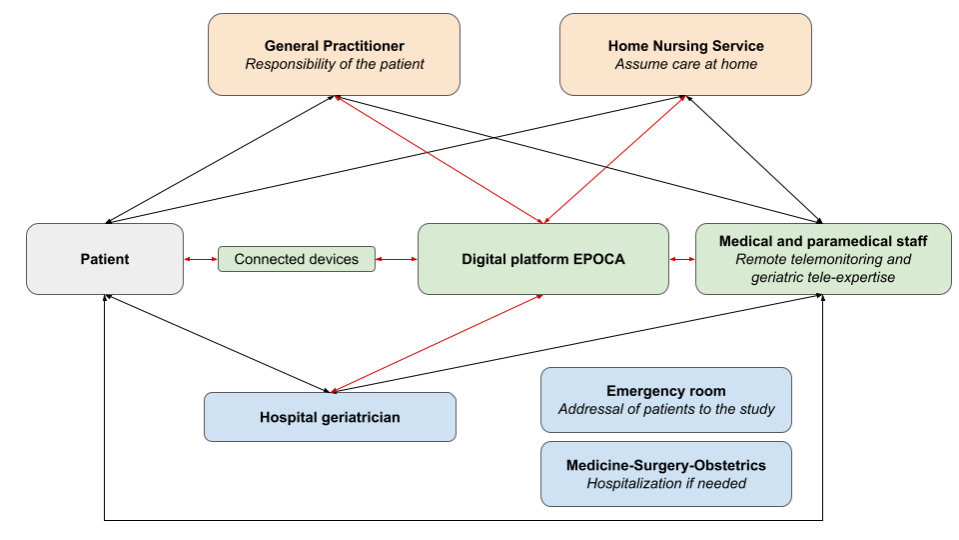
Collaboration between the EPOCA remote patient monitoring system and the health care professionals in the Vigie-Age project.

The Vigie-Age project is a collaborative multidisciplinary hospital-community initiative that started in 2019 in the Hauts-de-Seine department (Paris area, France) that aims to ensure comprehensive and coordinated care for older adults with polypathology, enhancing their quality of life and reducing avoidable hospitalizations. In this context, the EPOCA RPM system collaborates with the geriatric departments of 4 hospitals, GPs, and home care services in the community. The EPOCA RPM system is a human and connected telemedicine solution that offers secure home support for complex patients. It consists of a 24/7 telemonitoring platform that enables continuous remote monitoring as well as human assistance for emergency and geriatric expertise and coordination among the community paramedical teams, the GP, and the geriatrician to set up personalized care plans (Figure 1).

In essence, the EPOCA RPM system technology consists of two parts: (1) a remotely connected device (bracelet or medallion) for alert or health data transfer and (2) an RPM center with trained advisors (nurses and physicians) who contact patients and caregivers when necessary. Hospital geriatricians play a pivotal role in providing ongoing medical expertise through regular teleconsultations and tele-expertise while being in constant contact with the other health professionals involved in patient care plans. Self-employed nurses from community health care services conduct regular assessments and provide necessary home care.

This system is a medical device that meets the regulatory requirements for disease-specific telesurveillance pathways of remote medical telesurveillance operators. It also complies with the General Data Protection Regulation and health data hosting standards.

### Study Goals

This study aimed to investigate whether entering the EPOCA RPM system as part of a hospital-community coordination of care program is associated with a reduction in hospital admissions, including ED visits, and shorter hospital stay duration.

## Methods

### Study Design

A retrospective observational multicenter cohort study was conducted on patients benefiting from the EPOCA RPM system within the Vigie-Age project to ensure continuity of a long-term care, referred to as medical support at home (MSAH). The intensity of the intervention was adapted to the level of the participants’ disability.

The disability level was determined using the *groupe iso-ressources* (GIR). The GIR is a French administrative score system that assesses basic activities of daily living (walking, feeding, dressing, washing, and urinary and fecal continence) and more complex activities (perception of time and place, managing medication, managing shopping and finances, and using a telephone). The GIR score can vary from 1 (bed-ridden or major physical and mental limitations requiring assistance in all daily living activities) to 6 (no disability) [30]. Participants scoring 4, 5, or 6 on the GIR were considered as having no or moderate disabilities, whereas participants scoring 1, 2, or 3 on the GIR were considered to have severe disabilities.

Long-term MSAH started after the participant was discharged from one of the geriatric departments or the ED involved in the program or by home nursing professionals. Patients discharged from the ED could avoid an initial hospitalization as they were directly enrolled in the program. Every enrolled patient received an initial home visit, during which their needs and health status were thoroughly assessed. At this time point, comprehensive clinical, sociodemographic, and environmental data were recorded in the EPOCA RPM system. They also received teleconsultations and personalized installation of remote monitoring devices, as well as training and advice on the use of these devices. Multidisciplinary re-evaluations were systematically proposed at day 14, month 1, month 3, and then every 3 months. In case of an acute event, the home-based care can be temporarily intensified through acute geriatrics at home (AGH) program, to avoid hospitalization if possible. If hospitalization occurred in spite of this, AGH could reduce the duration of the hospitalization by anticipating hospital discharge. The long-term MSAH program could then continue after acute condition resolution.

### Inclusion and Exclusion Criteria

The criteria for admitting patients to MSAH follow-up were being aged ≥70 years and having polypathology (>2 affected systems). The participants did not enter the EPOCA RPM system simultaneously and so had very different durations of follow-up. All patients followed by the EPOCA RPM system in the MSAH long-term care pathway for at least 5 months between February 2022 and October 2023 were included in this study. We chose not to include participants with shorter follow-ups to obtain a median follow-up of close to 1 year and then better approximate the annual hospitalization rate.

### Ethical Considerations

This study was conducted in accordance with the MR-004 framework of the French research methodology guidelines. It was registered with the French data protection authority (Commission Nationale de l’Informatique et des Libertés) under declaration 2234081.

In compliance with national regulations, retrospective analyses of health data collected during routine care that comply with the MR-004 framework and for which patients have provided consent do not require ethics committee approval (equivalent to institutional review board approval) [31-33]. This exemption is explicitly supported by the Commission Nationale de l’Informatique et des Libertés guidance [31], which references the Ministerial Order of May 3, 2018 [32], and affirms that research protocols fully adhering to MR-004 provisions are not subject to ethical review by bodies such as the CEREES (Comité d’Expertise pour les Recherches, les Etudes et les Evaluations dans le domaine de la Santé), as stated in Title VIII of the official documentation.

All participants enrolled in this study received written information at the time of inclusion indicating that data collected in the EPOCA RPM system database regarding medical records and follow-up outcomes could be used retrospectively for research purposes. Participants also provided written informed consent for the use of their data after anonymization when they entered the EPOCA RPM system.

All data used in this study were fully anonymized before analysis to ensure confidentiality and privacy. No compensation was provided to participants.

### Data Collection and Data Storage

The medical and paramedical teams (internal or external to the EPOCA RPM system) in charge of each patient collected data and compiled them into the EPOCA RPM system database at baseline and during the follow-up.

The data consisted of sociodemographic and environmental information (age, gender, social isolation, presence of nonprofessional and professional caregivers, and being under legal protection), disability level according to the GIR score in activities of daily living [34] and instrumental activities of daily living [35], and cognitive status according to the Mini-Mental State Examination [36].

Polypathology was assessed using the global Charlson Comorbidity Index and the number of chronic conditions that each patient had out of a list of the pathologies frequently observed in this population. The number and type of molecules in usual medication was also considered. Nutritional status was evaluated using BMI and history of recent weight loss.

Social isolation was first assessed at baseline by nurses during home visits using three items: (1) living situation (living alone vs not living alone), (2) presence of a nonprofessional caregiver (yes vs no), and (3) presence of professional care at home (eg, daily nurse visits). On the basis of these items, social isolation was classified into 3 categories: none, geographic (physical isolation but with some social support), and total (living alone without informal or professional caregiving support). The social isolation category represents a combination of both geographic (physically separated) and total isolation.

### Primary Outcomes

A before-and-after pragmatic approach was used to compare the primary outcomes. These outcomes were the total number of hospital admissions, including ED visits; hospitalization rate per participant; and the cumulative duration of hospital stays in days for the total population and per patient. The average length of hospital stays (ie, the mean number of hospitalization days considering the overall cohort) was calculated by dividing the total number of hospitalization days of all patients during the period by the total number of hospitalizations.

Given the retrospective nature of the study and the availability of data collected over varying lengths of time, a proration approach was used to adjust measures on an annual basis for the period during which patients were under the EPOCA RPM system (Y period). The annualized rate of outcomes measured during this period was then compared to the outcomes measured during the year before the entry into the program (Y – 1 period). If participants ended the follow-up after 5 months for any reason, including death, the primary outcomes were collected until the date when the follow-up ended. Hospitalization data over the Y – 1 period were obtained from patient medical records.

### Statistical Analysis

Sociodemographic and clinical data were expressed using mean and SD or median and IQR for continuous variables and as number and proportion for categorical variables. Data were described for the entire population and compared according to disability level using Student 2-tailed *t* tests or Wilcoxon or chi-square tests.

The absolute and relative variations in number of hospitalizations and ED visits, cumulative length of hospital stay during the periods, and the average length of hospital stays before and after entry into the EPOCA RPM system were obtained subtracting the values measured during the previous year (Y – 1) from those of the EPOCA RPM follow-up year (Y), adjusted on an annual basis. The Shapiro-Wilk test was used to assess the normality of the data distribution. As the data were not normally distributed, a Wilcoxon test was used to compare outcomes on the year before (Y – 1) and after (Y) the intervention.

Statistical significance was considered when *P*<.05. All statistical analyses were conducted using Microsoft Visual Studio Code (version 1.84.2) in Python language (version 3.11.5; Python Software Foundation) using the *SciPy* and *statsmodels* packages.

A generalized linear model with a negative binomial distribution and a log-link function was used to account for overdispersion in the count outcome. Model selection was based on the theoretical relevance and statistical significance of predictors.

### Sample Size Calculation

A sample size calculation was conducted with an α risk of 1% and β risk of 5%, an estimated yearly duration of hospital stays of 13 days, and an expected decrease of 30%. These data were selected from a pilot study of MSAH patients with a mean follow-up duration of 5 months. A minimum of 111 patients was aimed for.

## Results

### Participant Characteristics

**Figure 2:**
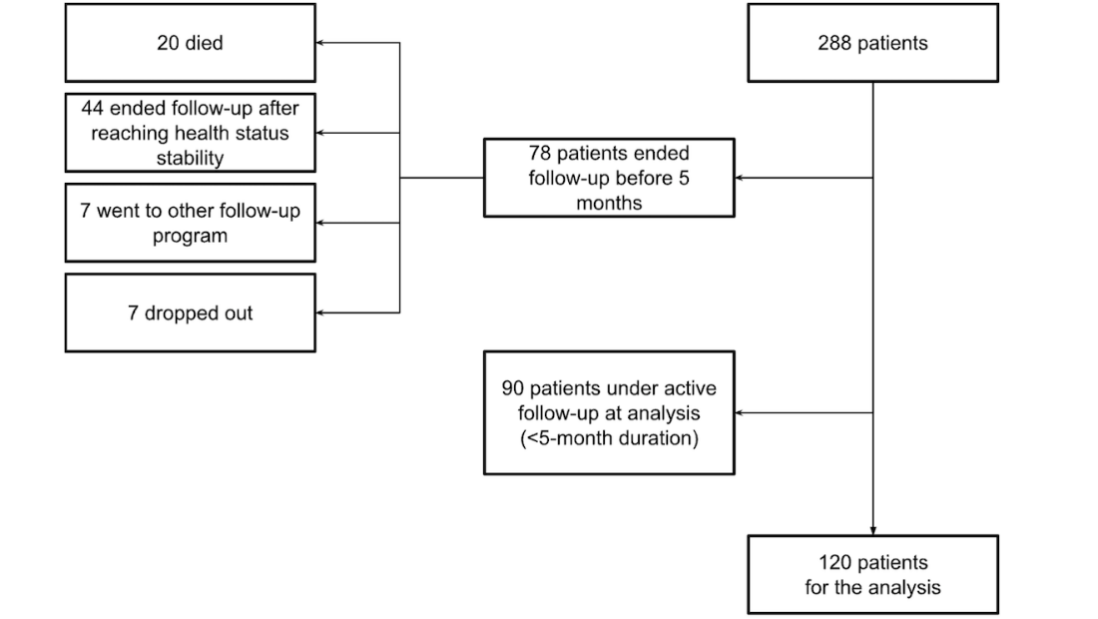
Flow chart of participant selection and the total number of patients included in the Vigie Age project.

This study included 120 participants (Figure 2; n=71, 59.2% were women, with a mean age of 86.8, SD 7.9 years); 57.5% (69/120) were considered as having no or moderate disability (GIR score of 4-6), and 42.5% (51/120) had severe disabilities (GIR score of 1-3). Table 1 summarizes the characteristics of the participants. Social isolation affected 43.3% (52/120) of the participants. People with a higher level of disability had a significantly higher number of external caregivers, were more frequently under legal guardianship, had lower Mini-Mental State Examination scores, and had a more frequent history of dementia. The mean number of chronic diseases was 6.1 (SD 2.5), and the mean number of medications taken was 8.8 (SD 3.4) without difference between disability level groups.

**Table 1:** Characteristics of the participants (N=120).

Variable			Participants with missing data, n		All, n/N (%)		Disability level, n/N (%)				*P* value
							No or moderate		Severe		
**Demographic data**											
	Age (y)	0		86.8 (7.9)^a^		86.7 (6.6)^a^		86.9 (9.4)^a^		.90	
	Gender (woman)	0		71/120 (59.2)		45/69 (65.5)		26/51 (51)		.17	
	Social isolation	0		52/120 (43.3)		33/69 (47.8)		19/51 (37.3)		.77	
	Presence of a nonprofessional caregiver	0		66/120 (55)		32/69 (46.4)		34/51 (66.7)		*.04* ^b^	
	Number of children	4		2.2 (1.8)^a^		1.8 (1.6)^a^		2.6 (2.2)^a^		*.002*	
	Under legal guardianship	11		23/109 (21)		8/63 (13)		15/46 (33)		*.02*	
**Professional caregiver**											
	Number of external caregivers	0		2.4 (1.7)^a^		1.9 (1.5)^a^		3.1 (1.6)^a^		*<.001*	
	Nurse	8		72/112 (64.3)		34/62 (55)		38/50 (76)		*.03*	
	Care assistant	14		67/106 (63.2)		31/59 (53)		36/47 (77)		*.02*	
	Physiotherapist	14		50/106 (47.2)		26/58 (45)		24/48 (50)		.74	
	Home nursing services	22		39/98 (39.8)		14/56 (25)		25/42 (59)		*.001*	
	Home care helper	21		38/99 (38.4)		11/53 (21)		27/46 (59)		*<.001*	
	Housekeeper	84		16/36 (44.4)		10/20 (50)		6/16 (37)		.68	
	Meal delivery	9		15/111 (13.5)		9/63 (14)		6/48 (12)		>.99	
	Declared GP^c^	0		9/120 (7.5)		4/69 (5.8)		5/51 (9.8)		.63	
**Functional and cognitive assessment**											
	GIR^d^ score (1-6)	0		3.4 (1.3)^a^		4.2 (0.98)^a^		2.3 (0.87)^a^		*<.001*	
	ADL^e^—Katz index – score (0-6)	10		3.6 (1.9)^a^		4.4 (1.5)^a^		2.4 (1.7)^a^		*<.001*	
	IADL^f^—Lawton index – score (0-8)	12		2.8 (2.3)^a^		4.0 (2.3)^a^		1.2 (0.8)^a^		*<.001*	
	MMSE^g^ - score (0-30)	57		21.8 (5.7)^a^		23.6 (4.1)^a^		18.9 (6.8)^a^		*.002*	
**Nutritional assessment**											
	BMI (kg/m^2^)	5		25.4 (5.8)^a^		25.3 (5.2)^a^		25.5 (6.7)^a^		.88	
	Abnormal weight loss	0		21/120 (17.5)		9/69 (13)		12/51 (23.5)		.21	
**Comorbidities**											
	Charlson Comorbidity Index – score (0 – 37)	0		7.4 (2.4)^a^		7.4 (2.3)^a^		7.3 (2.5)^a^		.84	
	Number of chronic diseases	0		6.1 (2.5)^a^		6.0 (2.5)^a^		6.3 (2.6)^a^		.42	
	Essential hypertension	0		89/120 (74.2)		50/69 (72.5)		30/51 (58.8)		.78	
	Heart failure	0		50/120 (41.7)		31/69 (44.9)		19/51 (37.3)		.51	
	Atrial fibrillation and flutter	0		43/120 (35.8)		26/69 (37.7)		17/51 (33.3)		.76	
	Coronary artery disease	0		21/120 (17.5)		15/69 (21.7)		6/51 (11.8)		.24	
	Dementia	0		43/120 (35.8)		18/69 (26.1)		25/51 (49)		*.02*	
	Gait disorders	0		42/120 (35)		29/69 (42)		13/51 (25.5)		.09	
	Depression	0		28/120 (23.3)		15/69 (21.7)		13/51 (25.5)		.79	
	Cerebrovascular accident	0		23/120 (19.2)		9/69 (13)		14/51 (27.5)		.08	
	Diabetes mellitus	0		20/120 (16.7)		10/69 (14.5)		10/51 (19.6)		.62	
	Chronic obstructive pulmonary disease	0		19/120 (15.8)		13/69 (18.8)		6/51 (11.8)		.43	
	Anemia	0		28/120 (23.3)		12/69 (17.4)		16/51 (31.4)		.12	
**Medications**											
	Number of medications	0		8.8 (3.4)^a^		8.8 (3.6)^a^		9.0 (3.1)^a^		.74	
	Antihypertensive drugs	0		91/120 (75.8)		54/69 (78.3)		37/51 (72.5)		.61	
	Anticoagulant drugs	0		55/120 (45.8)		33/69 (47.8)		22/51 (43.1)		.69	
	Antiaggregants	0		31/120 (25.8)		19/69 (27.5)		12/51 (23.5)		.78	
	Diuretic drugs	0		49/120 (40.8)		33/69 (47.8)		16/51 (31.4)		.10	
	Benzodiazepines	0		47/120 (39.2)		22/69 (31.9)		25/51 (49)		.09	
	Antidepressants	0		35/120 (29.2)		17/69 (24.6)		18/51 (35.3)		.29	

^a^Data are presented as mean (SD).

^b^Italics indicate statistical significance.

^c^GP: general practitioner.

^d^GIR: *groupe iso-ressources*.

^e^ADL: activity of daily living.

^f^IADL: instrumental ADL.

^g^MMSE: Mini-Mental State Examination.

### Overall Dropout Rate and Average Duration of Follow-Up

Among the 120 participants of this study, the duration of follow-up in the EPOCA RPM system ranged from 161 to 597 days, with a median of 323 (IQR 225-469) days. A total of 41.7% (50/120) of the patients were followed for >1 year. A total of 28.3% (34/120) of the patients ended the follow-up—12.5% (15/120) died, 12.5% (15/120) ended follow-up after reaching health status stability and the expected follow-up duration, 2.5% (3/120) dropped out, and 0.8% (1/120) went to another follow-up program.

### Hospitalization and ED Admission Rate and Hospital Stay Duration

Tables 2 and 3 present outcome variation between the Y and Y – 1 periods.

**Table 2:** Total hospitalization duration, number of hospitalizations and emergency department visits, and average length of stay in all populations during the Y and Y – 1 periods. (d): days.

			Y – 1 period		Y period		Change—Y versus Y – 1 (%)
**All (N=120)**							
	Total hospitalization duration (d)	3345		1248		−63	
	Number of hospitalizations	157		81		−48	
	Number of emergency department visits	166		86		−48	
	Average length of hospital stay (d)	21.3		15.4		−28	
**No or moderate disability (n=69)**							
	Total hospitalization duration (d)	1500		753		−50	
	Number of hospitalizations	83		47		−43	
	Number of emergency department visits	87		52		−40	
	Average length of hospital stay (d)	18.1		16.0		−12	
**Severe disability (n=51)**							
	Total hospitalization duration (d)	1845		495		−73	
	Number of hospitalizations	74		34		−54	
	Number of emergency department visits	79		34		−57	
	Average length of hospital stay (d)	24.9		14.6		−41	

**Table 3:** Hospitalization duration and number of hospitalizations and emergency department visits per patient during the Y and Y – 1 periods (N=120). (d): days.

Variable	Y – 1 period, median (IQR)				Y period, median (IQR)				*P* value		
	All	No or moderate disability (n=69)	Severe disability (n=51)	All		No or moderate disability (n=69)	Severe disability (n=51)	All		No or moderate disability (n=69)	Severe disability (n=51)
Total hospitalization duration (d)	14.0 (3.0-35.0)	13.0 (2.0-20.0)	17.0 (8.5-47.0)	0.0 (0.0-10.5)		0.0 (0.0-14.0)	0.0 (0.0-3.5)	*<.001* ^a^		*<.001*	*<.001*
Number of hospitalizations	1.0 (1.0-2.0)	1.0 (1.0-1.0)	1.0 (1.0-2.0)	0.0 (0.0-1.0)		0.0 (0.0-1.0)	0.0 (0.0-1.0)	*<.001*		*.002*	*<.001*
Number of emergency department visits	1.0 (1.0-2.0)	1.0 (1.0-2.0)	1.0 (1.0-2.0)	0.0 (0.0-1.0)		0.0 (0.0-1.0)	0.0 (0.0-1.0)	*<.001*		.003	*<.001*

^a^Italics indicate statistical significance.

In the total study population, all the indicators decreased between the Y – 1 and Y periods, including the total hospitalization duration (−63%), the number of hospitalizations (−48%), the number of ED visits (−48%), and the average length of hospital stays (−18%). The decrease was observed in the 2 subgroups by level of participant disability and was even greater in the participant group with more severe disabilities (Table 2).

During the Y – 1 period, 86.7% (104/120) of the participants had been hospitalized at least once. This proportion dropped to 37.5% (45/120) after entering the EPOCA RPM system (Y period). As shown in Table 3, the total hospitalization duration per patient, as well as the number of hospitalizations and ED visits per patient, decreased significantly between the Y – 1 and Y periods. It corresponds to a median reduction of 14 days spent at the hospital per patient per year. Hospitalization and ED visit rates per patient per year also decreased between the 2 periods. The decrease in indicators concerned the 2 groups of participants and was greater in participants with the highest level of disability (Table 3).

To identify predictive factors associated with the number of hospitalization days in the follow-up period (Y), we conducted a secondary analysis using a generalized linear model. The results, presented in Multimedia Appendix 1, identified several variables significantly linked to hospitalization duration (pseudo *R*^2^=0.55). For instance, compared to the woman gender, the man gender was linked to a significantly higher number of hospital days (β=1.4233; *P*<.001). Similarly, an increased number of chronic diseases (β=0.2388; *P*<.001), age (β=0.0441; *P*=.008), and number of treatments (β=0.1787; *P*<.001) were also significantly linked to a higher number of hospitalization days. In contrast, other variables such as number of hospitalizations during the Y – 1 period, number of external caregivers, social isolation, and GIR score did not show a significant association with hospitalization days during the Y period.

## Discussion

### Association of the EPOCA RPM System With Hospitalization and ED Visit Reductions

In this retrospective, real-life study based on the results of 120 patients monitored for a minimum period of 5 months (median duration of follow-up 323, IQR 225-469 days), we found that using RPM in the context of a coordinated hospital-community program was associated with an important reduction in number of hospitalizations and duration, as well as in ED visits. Even in cases in which clinical outcomes are comparable, avoiding an ED visit may be preferable from both a patient-centered and a health system perspective, reducing congestion, costs, and unnecessary admissions. Moreover, when hospitalization is necessary, direct admission to the appropriate inpatient unit bypassing the ED can further streamline care, improve patient experience, and optimize resource use. Similarly to our findings, previous studies have shown that telemedicine is associated with improved patient outcomes [13,21,28,37]. However, when compared to previously published promising results on hospital or ED admissions, the improvements in outcome measures obtained in our study were more pronounced than those reported in other studies [28,29,37]. The eCOBAHLT randomized controlled trial published in 2023 is the study that comes closest to ours in terms of population and RPM system [29]. However, we observed a greater reduction in hospitalization rate in our study, and while Tchalla et al [29] found an increase in ED visits in the intervention group, ED visits also decreased in our study. This potential positive effect on ED visits may result in a better coordination of care as the EPOCA RPM system was included in a more global hospital-community program. It is known that RPM may increase ED visits by increasing alerts generated via the monitoring of biometric parameters. However, in the eCOBAHLT randomized trial, the increase in ED visits was not associated with an increase in hospitalizations, suggesting that most of these ED visits may be related to health problems manageable through GPs and appropriate community care services.

As part of the Vigie-Age project, the EPOCA RPM system ensures better monitoring of health parameters but also a coordinated, rapid response, limiting hospitalization whenever possible. In case of an acute event, the intervention could be reinforced during a short period referred to as AGH to avoid hospitalization if possible. If hospitalization occurs in spite of this, the AGH could reduce the duration of hospitalization by anticipating hospital discharge. Thus, our study also found that the EPOCA RPM system was associated with a 6-day reduction in average duration of incident hospitalizations. Long durations of hospital stays in older adults with polypathology are an important issue for patients and the health care system, increasing the risk of hospital-acquired complications, loss of independence, and costs. As hospitalization is linked to functional decline in older adults [38], a decrease in hospital admissions may help in maintaining functional abilities. Although we did not measure the participants’ quality of life, telemonitoring has been associated with improved quality of life in older adults, especially in the physical domain of the 12-Item Short Form Health Survey [39]. Moreover, de Batlle et al [39] found that implementing RPM in older adults with polypathology was cost-effective. A specific study of the cost benefits of the use of the EPOCA RPM system is ongoing with access to the French administrative health care database. This study will analyze all health costs of participants years before inclusion in the program and while under the EPOCA RPM system care.

### Stronger Effect in Complex Participants

The study population of the eCOBAHLT trial is close to our study population in terms of age and comorbidities except for the proportion of participants with dementia, which constituted <2% in this previous study (compared to 43/120, 35.8% in our study). Cognitive disorders limit participation in randomized controlled trials for ethical reasons. However, it is imperative to evaluate the impact of RPM systems in populations with dementia as they are among the people most likely to benefit from them but also be less accepting of the intervention. Interestingly, the greater reduction in hospitalizations and ED visits was observed in more complex participants with a higher level of disability and cognitive decline. This suggests that RPM may be used beneficially for people with cognitive disorders with the support of home care services. It is important to consider RPM as a coordination tool that does not replace home care workers. For participants with more severe disabilities, personal home care services were frequently set up before the start of RPM. Our results suggest a good coordination between the EPOCA RPM staff and the personal home care services. Indeed, the EPOCA RPM system facilitates interactions among patients, their personal caregivers, and the health care system, minimizing the burden on patients while maximizing the effectiveness of remote monitoring. The multidisciplinary operational team not only adapts treatment but also deals with social issues and coordinates care alongside primary care professionals. This multidimensional approach contributes significantly to the positive results observed in our study even in participants with highest level of disability.

Confounding findings suggest that man gender, older age, polypharmacy, and multimorbidity are significant predictors of increased hospitalization days during follow-up, highlighting the burden of complex health profiles on health care use. In contrast, factors such as previous hospitalizations, social isolation, and functional dependency (as assessed using the GIR score) were not significantly associated with increased hospitalization days, which may reflect either measurement limitations or the predominance of clinical over social factors in determining hospitalization duration in these patients followed via EPOCA RPM.

### Patient and Caregiver Acceptance

Patient and caregiver acceptance of any health care intervention is a key factor for initial and long-term success. The implementation of RPM can be perceived as an additional constraint that can worsen the burdens of patients and their caregivers. Our data suggest a very good acceptability, as evidenced by the low dropout rate (3/120, 2.5% of patients from the study and 10/288, 3.5% of the entire cohort of patients), with only 1.8% (5/288) dropping out before 150 days (approximately 5 months) of follow-up.

### Study Limitations

There are some intrinsic limitations to our study that should be considered when interpreting the results. First, this study was based on retrospective data and, therefore, had the limitations of an observational study design. This study did not explicitly consider temporal variations in the use of the EPOCA RPM system. Over time, external factors, medical developments, introduction of additional interventions, and continuous improvement of the system may have had an influence on the results. As a retrospective study, the data might not be comprehensive. However, we believe that any underestimation regarding primary outcomes is more likely to impact the Y – 1 period.

Second, the effect of prorating the data over 1 year, which also represents itself a study limitation, was clearly attenuated by the average duration of patient follow-up (close to 1 year; median 323, IQR 225-469 days). However, for certain patients, only a specific period of the year was studied and prorated, such as the summer (or winter) period, which could have introduced a bias toward the avoidance of winter respiratory diseases and distort the analysis of variations in days of hospitalization. Conversely, including only the winter period could overestimate such hospitalizations. Observation over a more extended period could strengthen the hypothesis that MSAH monitoring supports a sustainable way of staying at home instead of requiring hospitalization.

Third, the size of the patient sample was small, and short follow-up durations (<5 months) were excluded from the analysis, which may introduce bias, although the sample size allowed us to observe statistically significant differences in the outcome measures.

Finally, there was no model of usual care used as a control group, allowing for concomitant comparison of the results. To address these limitations, a large-scale randomized controlled trial is set to begin in 2025 (NCT06845917) to generate more robust, high-level evidence.

### Conclusions

This retrospective observational study showed that home-based RPM could help reduce the total length of hospital stays and the total number of hospital admissions and ED visits. These results are similar to those of several previous studies evaluating the impact of telemedicine on patients who mostly had monopathology or were less complex. Our study adds to these previous reports by suggesting the similar effectiveness of telemedicine for highly complex patients with polypathology. In this specific population, the effectiveness of RPM relies not only on the monitoring of health indicators but also on a high level of responsiveness and coordination among multidisciplinary teams providing 24-7 human assistance, GPs, hospital geriatricians, and home care services. The costs of this enhanced system appear to be largely compensated by the significant reduction in hospitalization rates and emergency room visits but need to be confirmed through a cost-effectiveness study. Therefore, integrating the EPOCA RPM system into the health care system seems to offer more benefits than risks for older adults with polypathology and should help reduce health care costs.

## Appendix

Multimedia Appendix 1 Factors associated with number of hospitalizations days in Y period.

## Abbreviations

AGH: acute geriatrics at home
ED: emergency department
GIR: groupe iso-ressources
GP: general practitioner
MSAH: medical support at home
RPM: remote patient monitoring

## Data Availability

All data generated or analyzed during this study are included in this published article and its supplementary information files.
