# Supplementary material for: Impact of a Home-Based Remote Patient Monitoring System on Hospitalizations and Emergency Department Visits of Older Adults With Polypathology: Multicenter Retrospective Observational Study
Source: J Med Internet Res. 2025 Sep 10;27:e64989. doi: 10.2196/64989 (PMC12461160; doi:10.2196/64989)
Supplement: Multimedia Appendix 1 [file jmir_v27i1e64989_app1.docx]

## **Supplemental Material 1: Factor associated with number of hospitalizations days in Y period**

|  | **Coefficient (β)** | **SE^a^** | **z-value** | **p-value** | **95% CI^b^** |
| --- | --- | --- | --- | --- | --- |
| **Intercept** | -5.0201 | 1.580 | -3.178 | 0.001 | [-8.116, -1.924] |
| **Gender (male)** | 1.4233 | 0.247 | 5.762 | < 0.001 | [0.939, 1.908] |
| **Presence of a non-professional caregiver (yes)** | -0.5048 | 0.272 | -1.854 | 0.064 | [-1.038, 0.029] |
| **Social isolation (none)** | -0.1607 | 0.253 | -0.634 | 0.526 | [-0.657, 0.336] |
| **Age** | 0.0441 | 0.017 | 2.635 | 0.008 | [0.011, 0.077] |
| **GIR** | 0.0175 | 0.096 | 0.183 | 0.855 | [-0.170, 0.205] |
| **Number hospitalizations Y-1** | -0.0965 | 0.112 | -0.862 | 0.388 | [-0.316, 0.123] |
| **Number of chronic diseases** | 0.2388 | 0.051 | 4.725 | < 0.001 | [0.140, 0.338] |
| **Number of external caregivers** | -0.0476 | 0.079 | -0.601 | 0.548 | [-0.203, 0.108] |
| **Number of treatments** | 0.1787 | 0.038 | 4.720 | < 0.001 | [0.104, 0.253] |

## **Model:** Generalized Linear Model analysis results (GLM); Link function, Log; Pseudo R2: 0.55

## **^a^SE:** Standard error

## **^b^95 % CI:** Confidence interval. Values represent the 95% CI of the β coefficient
